# Trial Registration Numbers Are Underreported in Biomedical Publications

**DOI:** 10.1371/journal.pone.0049599

**Published:** 2012-11-14

**Authors:** Fleur T. van de Wetering, Rob J. P. M. Scholten, Tamara Haring, Michael Clarke, Lotty Hooft

**Affiliations:** 1 Dutch Cochrane Centre, Academic Medical Center, University of Amsterdam, Amsterdam, The Netherlands; 2 All Ireland Hub for Trials Methodology Research, Queen’s University of Belfast, Belfast, Northern Ireland; Johns Hopkins Bloomberg School of Public Health, United States of America

## Abstract

**Context:**

Since September 2005, the International Committee of Medical Journal Editors (ICMJE) has required that randomised controlled trials (RCTs) are prospectively registered in a publicly accessible database. After registration, a trial registration number (TRN) is assigned to each RCT, which should make it easier to identify future publications and cross-check published results with associated registry entries, as long as the unique identification number is reported in the article.

**Objective:**

Our primary objective was to evaluate the reporting of trial registration numbers in biomedical publications. Secondary objectives were to evaluate how many published RCTs had been registered and how many registered RCTs had resulted in a publication, using a sample of trials from the Netherlands Trials Register (NTR).

**Design, Setting:**

Two different samples of RCTs were examined: 1) RCTs published in November 2010 in core clinical journals identified in MEDLINE; 2) RCTs registered in the NTR with a latest expected end date of 31 August 2008.

**Results:**

Fifty-five percent (166/302) of the reports of RCTs found in MEDLINE and 60% (186/312) of the published reports of RCTs from the NTR cohort contained a TRN. In both samples, reporting of a TRN was more likely in RCTs published in ICMJE member journals as compared to non-ICMJE member journals (MEDLINE 58% vs. 45%; NTR: 70% vs. 49%). Thirty-nine percent of published RCTs in the MEDLINE sample appear not to have been registered, and 48% of RCTs registered in the NTR seemed not to have been published at least two years after the expected date for study completion.

**Conclusion:**

Our results show that further promotion and implementation of trial registration and accurate reporting of TRN is still needed. This might be helped by inclusion of the TRN as an item on the CONSORT checklist.

## Introduction

Guidelines and treatment recommendations rely on the findings of systematic reviews and meta-analyses of randomised controlled trials (RCTs). However, previous research has indicated that a significant proportion of healthcare research remains either unpublished, or published with different outcomes than originally intended [1]. Selective publication of RCTs, selective reporting of outcomes within RCTs and duplicate publication may distort the results of a RCT and, therefore, any systematic review that incorporate it. This could lead to inefficient care or the use of a harmful treatment for patients [1], [2].

To shed light on these sources of bias, prospective registration of RCTs at their inception in publicly accessible trial registries is advocated [3]. A prospective trial register contains both administrative and scientific information on each registered RCT [4]. A description of the proposed methodology for the RCT is documented in its trial registry record and could be cross-checked with the reported methods of a published RCT, to confirm that the trial was conducted and reported as intended^1^. Furthermore, if the results of a RCT have not been published, the responsible researchers could be contacted through information in the registry record and asked for details about the trial’s findings. Prospective trial registries thus create transparency, making it easier to identify published trial reports and to assess the risk of bias from selective publication.

Yet, there are also ethical reasons for registering RCTs. For example, participants in a RCT might expect that their contribution to research will be used to improve health care for other people. Open access to information about ongoing and completed trials will fulfill the ethical responsibility to the participants and should encourage greater trust in clinical research [5]. However, trial registries can only serve these goals when a registry record can easily be linked with its subsequent publication, or other source of the trial’s findings [6]. Hence, it is essential that all RCTs are registered and that their trial registration number (TRN) is included in all reports of their findings [2].

In 2004, the members of The International Committee of Medical Journal Editors (ICMJE) – a small working group of general medical journals – announced that they would only consider a RCT for publication after September 2005 if it had been registered before the enrollment of the first patient [7], [8]. In October 2011, 479 peer reviewed journals were listed as followers of the ICMJE’s Uniform Requirements for Manuscripts (URM) Submitted to Biomedical Journals. Although trial registration remains voluntary, the ICMJE’s registration policy has clearly contributed to a drastically increased number of registered trials [9]. However, trial registration by itself does not solve the problem of selective reporting and if trial registration numbers are not included in associated publications, comparisons between the published report and the registry record to identify of selective outcome reporting is made unnecessarily difficult.

In this study, we aimed to assess the adherence of biomedical journals to the trial registration policy from two different sides: from published reports after the completion of a trial and from the records in prospective registers before it began. The primary objective was to assess the reporting of trial registration numbers in biomedical publications. The secondary objectives were to evaluate how many published RCTs had been registered in prospective trial registries (in a register which is both a WHO Primary Registry and an ICMJE-approved registry [10]) and to assess how many of the sample of registered RCTs have been published at least two years after the intended date of study completion.

## Methods

We examined the reporting of trial registration numbers in two different samples of RCTs: one sample contained published reports of RCTs indexed in MEDLINE and the other contained RCTs registered in the Netherlands Trials Register (NTR). For both samples, a distinction was made between the reporting of trial registration numbers in reports published in journals that follow the ICMJE’s URM, compared to journals that do not explicitly follow this.

### MEDLINE Sample

PubMed (National Library of Medicine) was used to search MEDLINE. All indexed articles with publication type ‘randomized controlled trial’ published in November 2010 in core clinical journals and tagged as human studies were included. The subset limit core clinical journals restricts the search results to the 121 English language clinical journals formerly published as the Abridged Index Medicus. The following search strategy was used: randomized controlled trial[pt] AND (jsubsetaim[text] AND (“2010/11/01”[PDAT] : “2010/11/30”[PDAT])) [11].

For all identified RCTs, full articles were obtained and checked for trial registration numbers. If no registration number was identified in a report, we attempted to contact the corresponding authors to determine whether the RCT had been registered and if so, in which register. We limited our contact attempts to no more than two electronic mail messages. If we did not receive a response, we searched the WHO Search Portal, ClinicalTrials.gov of the US National Institutes of Health, International Standard Randomised Controlled Trial Number (ISRCTN) Register of Current Controlled Trials and relevant national trial registries (depending on the nationality of the main author) using the title, authors’ names, intervention and/or primary outcome [12]. If this searching failed to find a trial registry entry for the published RCT, we considered it as not being registered in a WHO Primary Registry or an ICMJE-approved registry [10].

We calculated the percentage of full articles from the MEDLINE sample in which a trial registration number was reported, doing separate calculations for journals following the ICMJE’s URM and those that do not follow this, based on the list available on the ICMJE website (ICMJE.org) in July 2011). In addition, we calculated the percentage of RCTs from the MEDLINE sample that had been registered in a WHO Primary Registry or an ICMJE-approved registry [10].

### Netherlands Trial Register Sample

For the second sample, we identified RCTs in the Netherlands Trial Register (NTR) in January 2010, which were marked as planned or ongoing at the time of their original registration and had a latest expected end date of 31 August 2008. Investigators who register their RCT in the NTR are requested to update their registry record annually to describe changes in the conduct or plans for the trial. We examined the registry records two years after their indicated end date, allowing sufficient time for investigators to amend their record if their planned end date had changed. We therefore assumed that all RCTs in the sample will have closed before our searches for publications of their findings.

We used the trial registration number to search MEDLINE in July 2011 for reports of the NTR trials. If no publication was identified, the MEDLINE search was expanded with details of the registry record title, name of the contact author, intervention and/or primary outcome. We also searched Google and Google Scholar (using the advanced Scholar search option) with the same search terms [13]. Identified articles were checked against information in the target trial’s entry in the NTR to determine if they were a correct match. If no publications were identified for a trial in the NTR, we attempted to contact the study’s investigators using information from within the NTR to determine if the trial had been published. We limited these attempts to no more than two electronic mail messages.

If no relevant articles were found and there was no response to our emails, we assumed that the results of the RCT had not been published. We obtained full copies of all articles found in MEDLINE, via Google or Google Scholar or from the investigators, and determined whether the trial registration number was reported, making a distinction between journals that follow the ICMJE’s URM and those that do not. When multiple publications of the same RCT were found, we included only one publication in our analysis, which if a RCT was reported in separate papers with and without a trial registration number, was the publication which included the trial registration number.

We also determined the percentage of registered RCTs that had been published by the time of our searches (i.e. at least two years after the intended date of study completion given in the NTR).

## Results

In MEDLINE, we identified 302 RCTs published in the core clinical journals in November 2010, of which 166 (55%) reported a trial registration number in the full text article. Separating the journals into those endorsing ICMJE URM and those that do not: 133 (58%) of the 229 RCTs published in the former and 33 (45%) of the 73 RCTs published in the latter reported a trial registration number (Table 1), although we found that 147 RCTs (64%) of the 229 RCTs published in an ICMJE URM journal had been registered (i.e. a further 14 trials). Of the 13 ICMJE working group member journals, we found reports of RCTs in six journals in November 2010, for a total of 39 RCTs. Of these, 38 (97%) reports included the trial registration number. Among the other journals that follow the ICMJE’s URM, we identified a total of 190 RCTs in 49 journals in November 2010. Of these, 95 (50%) reports included the trial registration number.

**Table 1:** Percentage of reported trial registration numbers and previous registration of trials in two samples of RCTs.

	ICMJE journal 1	Non-ICMJE journal	Total
**MEDLINE sample** 3	N = 229	n = 73	n = 302
Trial registration number reported	133/229 (58%)	33/73 (45%)	166/302 (55%)
Trial registered 2	147/229 (64%)	38/73 (52%)	185/302 (61%)
**NTR sample** 4	N = 162	n = 150	n = 312
Trial registration number reported	113/162 (70%)	73/150 (49%)	186/312 (60%)
**Total sample**	N = 391	n = 223	n = 614
Trial registration number reported	246/391 (63%)	106/223 (48%)	352/614 (57%)

1 International Committee of Medical Journal Editors.

2 RCTs registered in a WHO Primary Registry or an ICMJE-approved registry.

3 RCTs published in MEDLINE November 2010.

4 Published RCTs that were prospectively registered in the Netherlands Trial Registry (NTR).

We contacted the authors of the 136 RCTs without a trial registration number in their report, and 51 (38%) responded. This revealed that although 19 of these RCTs had been registered in a WHO Primary Registry or an ICMJE-approved registry, they had published their results without including their trial registration number. The other 32 RCTs had not been registered. We did not find any entry for the remaining 85 RCTs in the various trial registries that we searched and, therefore, we considered these RCTs as not having been registered. This means that of the 302 RCTs published in MEDLINE’s core clinical journals in November 2010, 117 (39%) had (apparently) not been registered.

In our search of the NTR in 2010, we identified 599 trial entries that indicated that they expected to end before 31 August 2008. Of these 599 RCTs, 312 (52%) had resulted in either one publication (252 RCTs) or two to four publications (60 RCTs). No publication was identified for 287 RCTs. We emailed the contact person for the study in the NTR entry to determine whether the trial had been published, and 45 (16%) responded. None of these stated that their trial had been published. Their answers indicated that 31 RCTs had been delayed and thus their publication is delayed, ten RCTs had been stopped early and their results had not (yet) been published, two RCTs had not started and will not result in a publication, and two RCTs were not delayed but had not yet been published. Consequently, it appears that 287 (48%) registered RCTs had not been published at least two years after study completion.

Of the 312 RCTs which had resulted in at least one publication, 186 (60%) included a trial registration number in at least one article. More than half of the published RCTs (162) had been published in a journal following the ICMJE’s URM, and 113 (70%) of these reported a trial registration number. A trial registration number was reported in 73 (49%) of the 150 RCTs published in non-ICMJE journals (Table 1).

Of the 60 registered RCTs that had been published in two or more articles, 24 (40%) did not declare their trial registration number in any of their multiple publications or were inconsistent in their reports.

None of the NTR trials were published during November 2010 and, so, there is no overlap between the MEDLINE and the NTR samples. Therefore, adding the two samples together, of the 614 publications of RCTs, 352 (57%) reported a trial registration number (Table 1). A trial registration number was reported in 246/391 (63%) RCTs that had been published in journals following the ICMJE’s URM and in 106/223 (48%) RCTs published in non-ICMJE member journals.

## Discussion

We have shown that many recently conducted RCTs do not report a trial registration number in their publication (43%), have not been registered (39%), or have not published their findings at least two years after the intended date of study completion (48%).

With respect to the reporting of trial identification numbers and previous registration, journals following the ICMJE’s URM are more likely to include these numbers in reports of RCTs than those that do not. The ICMJE’s policy, however, is to consider a RCT for publication only if it has been registered at inception in a publicly accessible trial registry and to report the assigned trial registration number in their publication (preferably at the end of the abstract). Therefore, not all journals following the ICMJE’s URM adhere to this policy: with one-third of the trials published in these journals in November 2010 appearing not to have been registered. We also found that 40% of the registered RCTs that resulted in two or more publications did not consistently mention their trial registration number in each publication. This lack of consistent reporting hampers easy identification of multiple or duplicate reports of the same RCT.

Our findings are consistent with previous research using different samples of trials, that has also shown low rates of trial registration, trial publication and/or underreporting of registration details in related reports [14], [15]. However, to our knowledge, this is the first study to approach the issue of registration and reporting simultaneously from two different sides: from published reports after the completion of a trial and from the records in prospective registers before it began.

With respect to our findings on previous registration, several factors may have influenced our findings in different directions. Firstly, despite our efforts to contact investigators by e-mail, there was poor response rate (38%) to our inquiries about registration (to authors in the MEDLINE sample) and about publication (to investigators in the NTR sample) (16%). This could have led to an overestimate of the number of RCTs classified as not having been registered and not being published (since non responses were regarded as not registered or not published, respectively). Secondly, we found that searching for records of published RCTs (in the absence of a registration number) in trial registries is challenging. We may have missed some trial registry records of eligible RCTs and, therefore, have underestimated the number of registered RCTs. Thirdly, when multiple publications of the same trial were identified in which only one of the publications reported a trial registration number, we included the publication which did declare a registration number. This possibly has led to an overestimation of the proportion of reports of RCTs which include a registration number.

However, these limitations are insufficient to render our general findings and conclusions invalid.

In summary, we have shown that a substantial proportion of RCTs is still being published without the reporting of a trial registration number, thereby weakening the ability of users of this research to identify multiple publications of the same RCT, to cross-check the published report with the original design of the study, or to assess the risk of bias from selective reporting^1^. Therefore, there is a continuing need to promote accurate reporting of trial registration numbers and, more fundamentally, to encourage trial registration itself. This might be helped by the addition of the trial registration number as an item to the CONSORT checklist; and we have suggested this to the group responsible for CONSORT. Authors and journal editors also have an important role and should pay more attention to accurate reporting of trial registration numbers, in order to make better use of the intended positive effects of prospective trial registration.
